# Supplementary material for: Protocol for Cell Colonization and Comprehensive Monitoring of Osteogenic Differentiation in 3D Scaffolds Using Biochemical Assays and Multiphoton Imaging
Source: Int J Mol Sci. 2023 Feb 3;24(3):2999. doi: 10.3390/ijms24032999 (PMC9917811; doi:10.3390/ijms24032999)
Supplement: Supplementary file 1 [file ijms-24-02999-s001.zip › Supplementary Materials (legends).docx]

**Supplementary Materials**

1. **Figures**

These figures show multiphoton images from SHG (red) and AF signals recorded as described in the Materials and Methods section. The mosaic images have an overlap between images of 10 % and represent sum intensity images from two z-planes (image distance ∆z=2.5 µm).

**Figure S1:** Comparison of collagen-I fiber networks shown without and with cells in α-TCP/HA and baghdadite scaffolds (osteogenic vs. normal growth conditions).

This figure compares the collagen-I networks (SHG) without and with AF from nearby cells in α-TCP/HA and baghdadite scaffolds under osteogenic vs. normal growth conditions. Upper row: SHG images (as in Fig. 7) showing only the various collagen-I networks. Lower row: Dual-channel SHG/AF images of the same (or slightly larger) regions as in upper row showing collagen-I together with the AF from cells. The AF signals highlights cell distribution, morphology and orientation (relative to the fiber networks), which appears clearly different under osteogenic compared to normal growth conditions. AF signals also derive from the scaffold (periphery of image I and II). Bright autofluorescent spots in III (upper left) and IV (bottom) may derive from baghdadite particles, but may eventually also derive from condensed dead cells, which often exhibit higher AF. Size bars: 100 µm.

**Figure S2:** Image collection showing collagen-I and cells in α-TCP/HA scaffolds (osteogenic vs. normal growth conditions).

Collection of images showing collagen-I networks (red) and AF signal (green) in α-TCP/HA scaffolds under osteogenic vs. normal growth conditions. Images are shown from both partially and completely filled pores. Single image snapshots are presented at the left (size bars: 50 µm). The collagen networks confirm the observations from Figure 7. Under osteogenic conditions (upper row), the fibers are clearly thickened into bundles and locally show a high degree of orientation (especially in the second image from left). There, it also becomes clear how the cells (green) arrange in stretched morphology corresponding to the fibers. This appearance seems to represent an advanced state of osteogenesis. Large-area mosaics are shown at the right (size bars: 100 µm). In these images, details of the fibers are more difficult to recognize, but they provide an overview of the tissue formation including the scaffold borders. There are remarkable regions in both single images and mosaics, in which SHG signals are completely absent (only AF signal, green).

**Figure S3:** Image collection showing collagen-I and cells in baghdadite scaffolds (osteogenic vs. normal growth conditions).

Collection of images showing collagen-I networks (SHG, red) and AF signal (green) in baghdadite scaffolds under osteogenic vs. normal growth conditions. Images are presented from both partially and completely filled pores. Single images are shown to the left (size bars: 50 µm) and mosaics to the right side (100 µm). Overall, under normal growth conditions (lower row), there appears to be little collagen-I fiberization (red) in many regions. Under osteogenic conditions (upper row), thick, highly assembled and partly wavy fiber bundles were frequently observed (see single images), which are arranged in characteristic patterns and can be seen impressively in large distribution in the mosaic image on the right. In the free spaces of these grid-like fibril patterns, cells can be recognized in a regular arrangement by their AF signal. AF signal from baghdadite struts was not observed.

1. **Videos**

The videos (.avi format) show multiphoton image z-stacks of cell-seeded α-TCP/HA and baghdadite scaffolds In all videos, image size was 200 x 200 µm, resolution was 1024 x 1024 pixels and z-distance (∆z) between images was 5 µm.

**A. Imaging of cell distribution in scaffold pores:**

The videos show z-stacks of cell-seeded α-TCP/HA and baghdadite scaffolds grown for three weeks in normal growth medium. The cells were fixed and co-stained for nuclei (DAPI) and actin cytoskeleton filaments (PF546-phalloidin). Laser excitation occurred at 775 nm, emissions were separated using a dichroic mirror at 560 nm. PF546 fluorescence was blocked in addition by a 620/60 bandpass filter. Numbers in upper right position represent the depth of the image within the stack.

**Video S1:** Concentric cell arrangement in partially filled scaffold pore.

The 3D image stack shows how the cells are arranged concentrically around the empty interior of a partially filled pore of a α-TCP/HA scaffold. Frame rate: 3 fps.

**Video S2:** Loose cell arrangement to multilayers in scaffold pore.

The stack shows the rather loose arrangement of cells within a 3D cell multilayer in a pore of a baghdadite scaffold. Frame rate: 2 fps.

**B. Imaging of hydroxyapatite (HA) distribution:**

The videos show z-stacks of cell-seeded α-TCP/HA and baghdadite scaffolds grown in normal growth and osteogenic conditions as described in Figure 8 of the manuscript. Cells were fixed and stained for HA crystals using the OsteoImage labeling kit. Excitation occurred at 775 nm, the signal range was from 500 to 550 nm. This excluded SHG signal, but included AF from cells, which was less bright, but in most cases showed a typical pattern that enabled identification of cells and their morphology. Numbers in upper right position represent the depth of the image within the stack. The frame rate of the videos is 5 fps.

**Video S3:** Cells in α-TCP/HA scaffold cultured under normal growth conditions.

After one third of the sequence, a high cell density is present and isolated cells with pronounced cell extensions and stronger AF signal can be detected. Hydroxyapatite crystals are almost undetectable. Imaging depth: 105 µm.

**Video S4:** Cells in α-TCP/HA scaffold cultured under osteogenic conditions.

Comparatively little AF originates from the mostly elongated cells, but the cells can be recognized. The hydroxyapatite crystals are very prominent, vary greatly in size and are well visible over the entire imaging depth. They appear to be both linearly aligned as thread-like structures (especially at the beginning of the sequence) and localized in close proximity to the cells (in the middle of the sequence). Imaging depth: 205 µm.

**Video S5:** Cells in baghdadite scaffold cultured under normal growth conditions.

Cells appear undefined (based on their AF signal) and only at a low density. A minor number of hydroxyapatite crystals is present. No thread-like structures are visible. Imaging depth: 205 µm.

**Video S6:** Cells in baghdadite scaffold cultured under osteogenic conditions.

Cells appear roundish. Hydroxyapatite crystals are present with variable shapes and evenly distributed. No thread-like structures are visible. Imaging depth: 185 µm.
