# Supplementary figures and images for: Protocol for Cell Colonization and Comprehensive Monitoring of Osteogenic Differentiation in 3D Scaffolds Using Biochemical Assays and Multiphoton Imaging
Source: Int J Mol Sci. 2023 Feb 3;24(3):2999. doi: 10.3390/ijms24032999 (PMC9917811; doi:10.3390/ijms24032999)

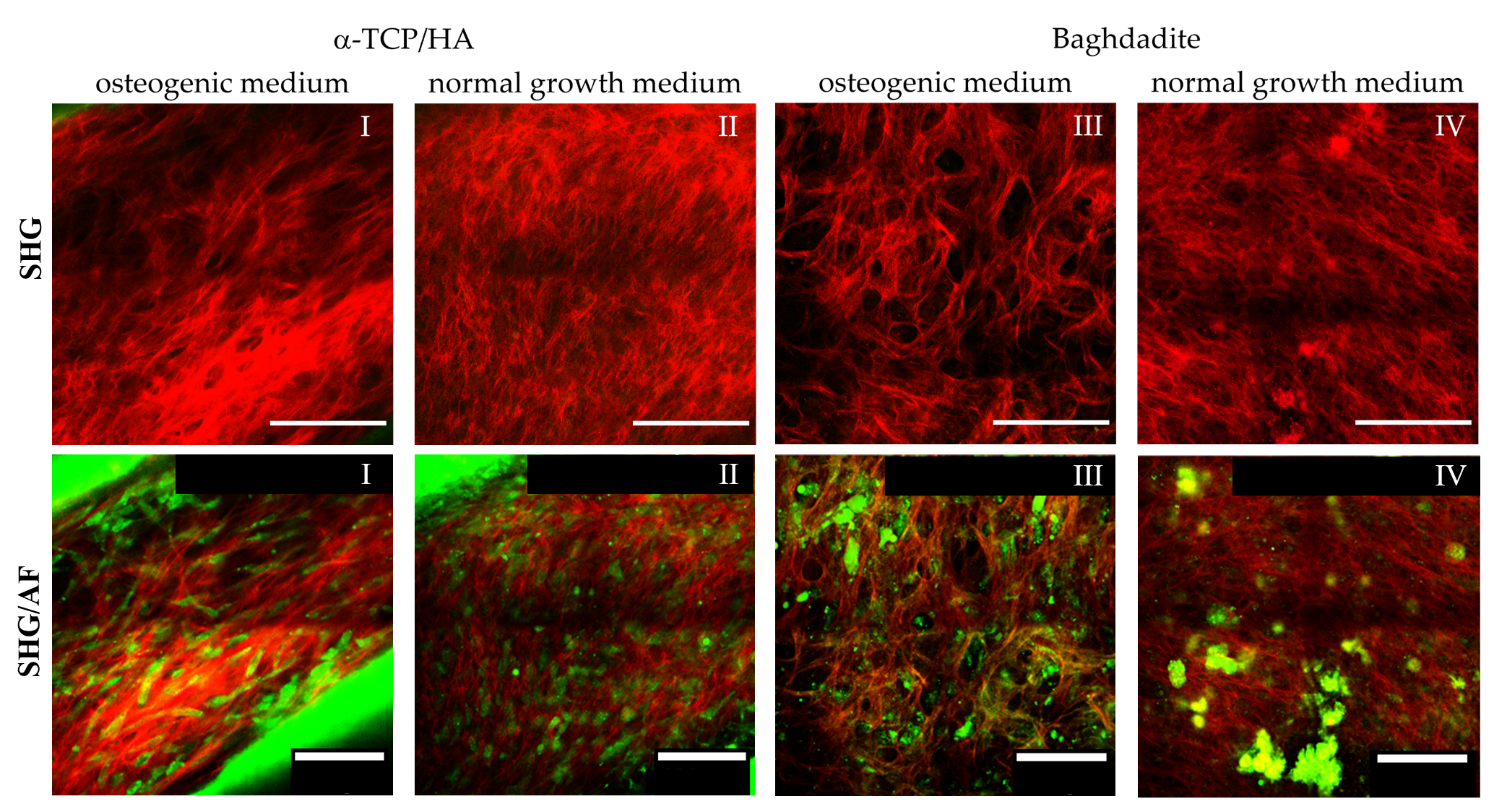

Supplement: Supplementary file 1 [file ijms-24-02999-s001.zip › Figure S1.png]

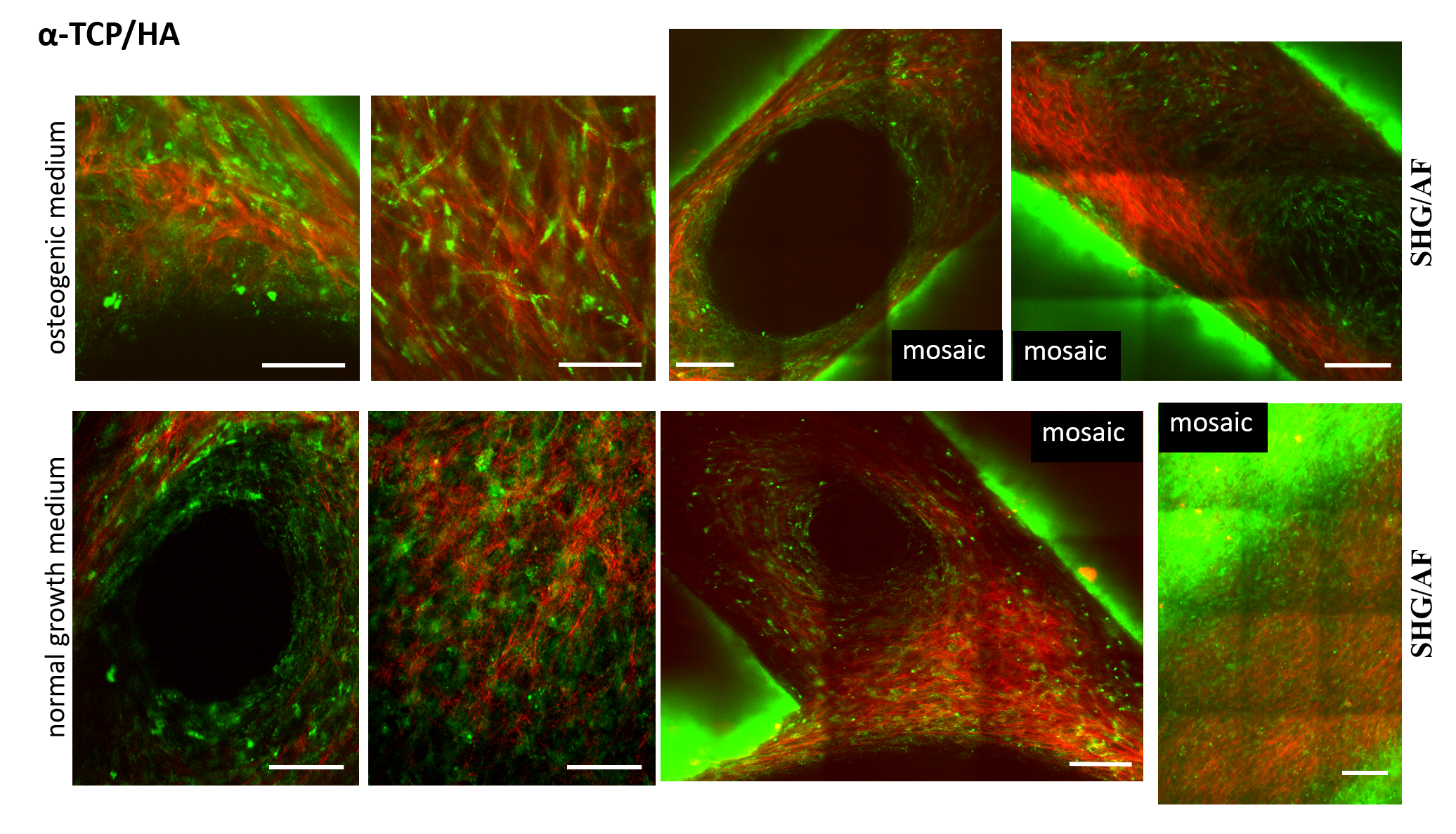

Supplement: Supplementary file 1 [file ijms-24-02999-s001.zip › Figure S2.png]

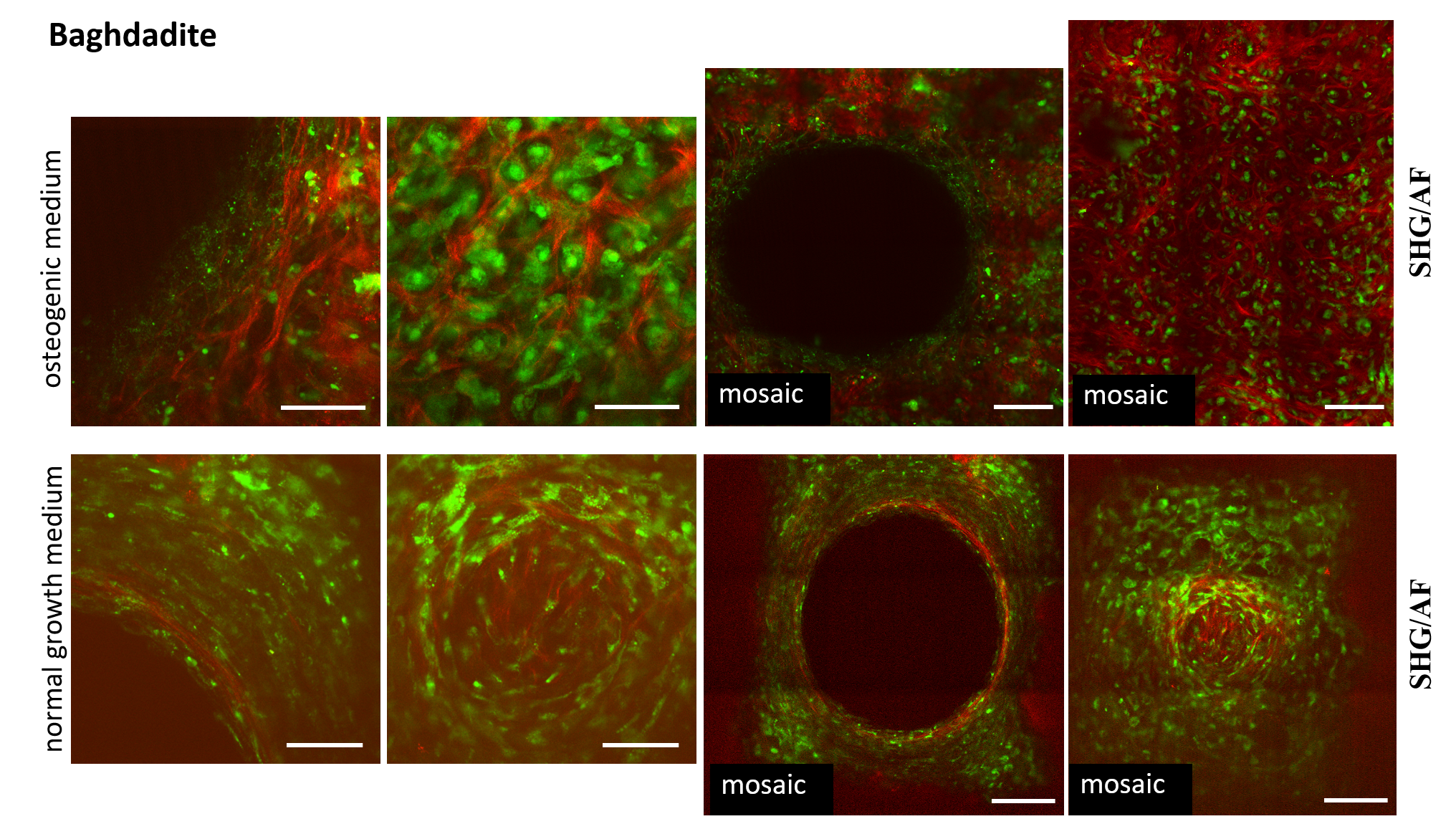

Supplement: Supplementary file 1 [file ijms-24-02999-s001.zip › Figure S3.png]
